# Vitamin D Receptor (*VDR*) Genetic Variants: Relationship of *FokI* Genotypes with *VDR* Expression and Clinical Disease Activity in Systemic Lupus Erythematosus Patients

**DOI:** 10.3390/genes13112016

**Published:** 2022-11-03

**Authors:** Mónica R. Meza-Meza, Barbara Vizmanos, Melissa Rivera-Escoto, Adolfo I. Ruiz-Ballesteros, Karen Pesqueda-Cendejas, Isela Parra-Rojas, Margarita Montoya-Buelna, Sonia Luquín, Bertha Campos-López, Paulina E. Mora-García, Sergio Cerpa-Cruz, Ulises De la Cruz-Mosso

**Affiliations:** 1 Red de Inmunonutrición y Genómica Nutricional en las Enfermedades Autoinmunes, Centro Universitario de Ciencias de la Salud, Universidad de Guadalajara, Guadalajara 44340, Mexico; 2 Instituto de Neurociencias Traslacionales, Departamento de Neurociencias, Centro Universitario de Ciencias de la Salud, Universidad de Guadalajara, Guadalajara 44340, Mexico; 3 Laboratorio de Evaluación del Estado Nutricio, Departamento de Clínicas de la Reproducción Humana, Crecimiento y Desarrollo Infantil, Centro Universitario de Ciencias de la Salud, Universidad de Guadalajara, Guadalajara 44340, Mexico; 4 Laboratorio de Investigación en Obesidad y Diabetes, Facultad de Ciencias Químico-Biológicas, Universidad Autónoma de Guerrero, Chilpancingo de los Bravo 39087, Mexico; 5 Laboratorio de Inmunología, Centro Universitario de Ciencias de la Salud, Universidad de Guadalajara, Guadalajara 44340, Mexico; 6 Departamento de Reumatología, O.P.D. Hospital Civil de Guadalajara Fray Antonio Alcalde, Guadalajara 44280, Mexico

**Keywords:** *VDR*, polymorphism, *FokI*, *BsmI*, *ApaI*, *TaqI*, vitamin D, calcidiol, calcitriol

## Abstract

Vitamin D (VD) deficiency is more frequent in systemic lupus erythematosus (SLE) patients than in control subjects (CS); genetic variants in the VD receptor (*VDR*) could contribute to the clinical disease activity. This study was aimed to determine the association of the *VDR* variants *FokI* (rs2228570), *BsmI* (rs1544410), *ApaI* (rs7975232), and *TaqI* (rs731236) with susceptibility to the disease, VD status, *VDR* mRNA expression, and clinical disease activity in SLE patients. A cross-sectional study was conducted in 194 SLE and 196 CS Mexican women. Immunoassays quantified serum calcidiol and calcitriol. Genotyping was performed by allelic discrimination assays and mRNA *VDR* expression by qPCR. The *FokI* variant was not in linkage disequilibrium with *BsmI*, *ApaI,* and *TaqI VDR* variants. SLE patient carriers of the TT *FokI* genotype showed higher clinical disease activity scores. Notably, the mRNA *VDR* expression was higher in SLE patients vs. CS, in active vs. inactive SLE patients, and in participants of both study groups with vitamin D deficiency, higher calcitriol levels, and TT *FokI* genotype carriers. In conclusion, the TT *FokI VDR* genotype was related to high *VDR* expression and clinical disease activity in systemic lupus erythematosus patients.

## 1. Introduction

Systemic lupus erythematosus (SLE) is a multisystem autoimmune disease characterized by immune regulation disrupted, leading to the production of antibodies against nuclear, cytoplasmic, or cell surface self-antigens, chronic inflammation, tissue, and organ damage [1,2,3]. Genetics, epigenetics, hormones, and environmental factors have been associated with SLE pathogenesis [4,5], and the association between specific phenotypes and variants of genetic loci has been identified [6]. SLE susceptibility can be influenced by the individual accumulation of risk alleles triggered by environmental factors, which could explain the discrepancies in SLE susceptibility between different populations [7]. The rate of SLE concordance in monozygotic twins is from 24 to 35%, compared between 2% and 5% in dizygotic twin pairs [8]. The genetic variability so far identified accounts for less than half of the SLE heritability, with modest overall effect sizes (OR~1.2 to 1.5) [6], and familial aggregation studies reported that between 10% and 12% of SLE patients have first- or second-degree family members with the disease, compared to <1% of controls [8].

Among the environmental risk factors for SLE is vitamin D deficiency, which is more frequent in SLE patients compared to the general population. It has been associated with susceptibility to several autoimmune diseases [3,5]. Moreover, it is related with clinical disease activity and organ damage [3,9]. The active form of vitamin D, calcitriol, is an immunomodulatory nutrient that participates in the control of self-tolerance, the production of cytokines, and the differentiation of immune system cells by its genomic pathway through the vitamin D receptor (VDR) [10,11,12,13].

VDR is a transcription factor regulated by ligand binding; it is located mainly in the cell nucleus and cytoplasm, where it is translocated into the nucleus after interaction with calcitriol. It is expressed in various organs involved in calcium metabolism and the immune and nervous system cells [12,14,15]. VDR can mediate the inhibition or induction of the transcription of vitamin D target genes by binding to vitamin D response elements (VDRE) in the DNA [12,16]. 

The *VDR* gene is located on 12q13.11 Crh. and it comprises 14 exons. Exon 1 is in the promoter region and has six variants (a–f), and exons 2–9 are in the coding region [1,14,17,18]. Four single nucleotide variants (SNVs) have been described in the *VDR* gene that may influence receptor structure and modulation of the response to vitamin D. These are named after the restriction endonuclease that were identified as *FokI* [rs2228570, 12:47879112 (GRCh38), C > T or also called F > f] located in exon 2; *BsmI* [rs1544410, 12:47846052 (GRCh38), A > G or B > b] and *ApaI* [rs7975232, 12:47845054 (GRCh38), A > C or A > a] both located in intron 8, and *TaqI* [rs731236, 12:47844974 (GRCh38), C > T or T > t] found in exon 9 [1,14,17,19].

According to their location, the *VDR* genetic variants can affect both the mRNA stability and the *VDR* gene transcription rate. These SNVs could change the expression, length, and activity of the protein associated with the susceptibility or severity of the SLE [1,20]. The presence of the C allele of the *FokI* variant has been described as being associated with a shorter VDR protein isoform of 424 amino acids, which is more transcriptionally active [21] since it interacts more efficiently with the transcription factor TFIIB [22], compared to the more extended variant of 427 amino acids (T allele). The possible effect of the *BsmI* and *ApaI* variants, due to their location in the same intron, may be an alteration in the splice sites for mRNA transcription or a change in the regulatory elements of the *VDR* intron. *TaqI*, on the other hand, does not change the length of the protein and results in a silent mutation [1,14,17,19].

*VDR* expression, apart from genetic factors, is also regulated at the transcriptional level by various hormones, including the parathyroid hormone (PTH), all trans-retinoic acid (atRA), and also by its ligand, calcitriol [23], since multiple enhancers in the *VDR* gene locus that can contribute to the *VDR* expression induced by calcitriol has been described [24]. Despite this evidence, mechanisms responsible for *VDR* expression still must be clarified [23], as well as their influence on the course of SLE. This study aimed to determine the association of the *VDR* variants *FokI* (rs2228570), *BsmI* (rs1544410), *ApaI* (rs7975232), and *TaqI* (rs731236) with susceptibility to the disease, vitamin D serum status, *VDR* mRNA expression, and clinical disease activity in SLE patients. 

## 2. Materials and Methods

### 2.1. Subjects

A comparative cross-sectional study was conducted in a population of 390 unrelated Mexican-Mestizo women to form two groups. The first group consisted of 194 SLE patients classified according to the 1997 American College of Rheumatology (ACR) criteria for SLE [25] recruited from 2017–2020 from the Rheumatology Department of the Hospital Civil Fray Antonio Alcalde, Guadalajara, Jalisco, Mexico. All SLE patients included in the present study had no recent infections, trauma, surgery, pregnancy, or other autoimmune systemic conditions unrelated to the SLE.

The second study group was formed with 196 women control subjects (CS). This CS population did not suffer from recent infections, trauma, surgery, pregnancy, or autoimmune conditions; additionally, they were asked about the presence of autoimmune diseases in their families, and they did not disclose that their close relatives, such as siblings, parents, and grandparents, had autoimmune diseases. 

All SLE and CS participants were classified as a Mexican-Mestizo population with a family history of ancestors, at least back to the third generation, in the same geographical area from Mexico [26].

### 2.2. Ethical Considerations

Informed written consent was obtained from all patients and subjects before enrollment in the study. The study was conducted in agreement with the International Ethical Guidelines for Health-related Research Involving Humans of the Council for International Organizations of Medical Sciences (CIOMS). The Research Ethical Committee of the Centro Universitario de Ciencias de la Salud, Universidad de Guadalajara (C.I. 03419 CUCS-UdG) and the Research Ethical Committee of the Hospital Civil Fray Antonio Alcalde (No. 280/19) approved the protocol.

### 2.3. Clinical Measurements

Demographic and clinical characteristics of participants were recorded, including age, gender, disease duration, pharmacotherapy received, clinical disease activity, and disease damage. The clinical disease activity was assessed by the Mexican Systemic Lupus Erythematosus Disease Activity Index (Mex-SLEDAI) [27] and the disease organ damage by the Systemic Lupus International Collaborating Clinics/American College of Rheumatology damage index (SLICC/ACR-DI) [28]. 

Additionally, the glomerular filtration rate (GFR) was estimated with the Chronic Kidney Disease Epidemiology Collaboration (CKD-EPI) equation based on serum creatinine (mg/dL) and using the parameters of sex, race, and age, expressed in mL/min/1.73 m^2^ of the body surface area [28], and was classified by the Kidney Disease: Improving Global Outcomes 2012 Clinical Practice Guideline (KDIGO 2012) categories, similar to that used in a previous study by the research group [3].

### 2.4. Quantification of Vitamin D Metabolites

A blood sample was obtained from each patient from antecubital venipuncture after an overnight fast (12 h) and then centrifuged for 10 min to attain the serum. Calcidiol and calcitriol serum levels were determined using an ELISA plate reader (Multiskan GO, Thermo Scientific™ 51119000, Walthman, MA, USA) with commercial competitive ELISA assays. For the quantification of calcidiol (25-hydroxy-vitamin D), the human soluble 25-OH Vitamin D ELISA Kit (detection limit of 1.6 ng/mL, Eagle Biosciences^®^, VID31-K01, Amherst, NH, USA) was used, and for calcitriol (1,25α-dihydroxy-vitamin D_3_), the human soluble 1,25α(OH)_2_D_3_ ELISA kit (sensitivity < 0.10 pg/mL, Cusabio^®^, CSB-E0512h, Wuhan, P.R., China) was used, all according to the manufacturer’s instructions.

### 2.5. Classification Criteria and Definitions

The reference cut-off values for interpreting serum calcidiol levels were the same as those used in our previous study of SLE patients, according to the cut-off values reported [3].

For calcitriol, we stratified its levels into tertiles: (a) low calcitriol serum levels = T1st (0.33 to ≤33.9 pg/mL), (b) average calcitriol serum levels = T2nd (>33.9 to ≤48.1 pg/mL), and (c) high calcitriol serum levels = T3rd (>48.1 to 157.3 pg/mL).

To estimate the vitamin D hydroxylation efficiency, we calculated the calcitriol/calcidiol ratio based on values of calcitriol (pg/mL) and calcidiol (ng/mL), which resulted in arbitrary units (pg/ng) that should indicate how many pg of calcitriol is produced per ng of circulating calcidiol [3]. The calcitriol/calcidiol ratio score was also stratified into tertiles: (a) low conversion rate of calcidiol to calcitriol = T1st (0.01 to ≤1.39 pg/ng), (b) average conversion rate of calcidiol to calcitriol = T2nd (>1.39 to ≤2.26 pg/ng), and (c) high conversion rate of calcidiol to calcitriol = T3rd (>2.26 to 23.6 pg/ng).

### 2.6. Genotyping of the VDR Genetic Variants

Total genomic DNA (gDNA) was isolated from peripheral blood leukocytes by the salting-out method [29]. Allelic discrimination assays carried out genotyping. The polymerase chain reaction (PCR) experiments were carried out in Light Cycler 8-Tube Strips (white) set for 96 reactions (Roche Diagnostics, Indianapolis, IN, USA) on the LightCycler^®^ 96 real-time PCR system (Roche Diagnostics, Indianapolis, IN, USA) as follows: 95 °C for 10 min, followed by 40 cycles of 95 °C for 15 s and 60 °C for 1 min. The *FokI* (C > T, rs2228570, C__12060045_20), *BsmI* (A > G, rs1544410, C___8716062_20), *ApaI* (A > C, rs7975232, C__28977635_10), and *TaqI* (C > T, rs731236, C___2404008_10) were identified using TaqMan^TM^ pre-designed genotyping assays (Applied Biosystems; Foster City, CA, USA). We randomly selected the samples in the subset for duplicate genotyping to confirm the genotypes of the four *VDR* genetic variants. We also used reference controls for each genotype assessed (VIC homozygous, FAM homozygous, and VIC/FAM heterozygous).

### 2.7. mRNA Expression of the VDR Gene

Total leukocytes were extracted from peripheral blood using density gradients (Lymphoprep™ H1077, 1077 g/mL, and Histopaque^®^ H1119, 1119 g/mL). Total RNA was obtained from peripheral blood leukocytes of SLE patients and CS by using the Chomczynski and Sacchi method [30], and 1 μg was used for the cDNA synthesis. The cDNA synthesis was performed with oligo (dT) primers (Oligo (dT)15 Primer, C1101, PROMEGA Corporation, Fitchburg, WI, USA). The mRNA expression of *VDR* was determined by the Taqman^TM^ qPCR method (Hs00172113_m1, Applied Biosystems; Foster City, CA, USA). The PCR reaction was performed on a LightCycler Nano System (Roche Applied Science, Penzberg, Germany) using the conditions indicated in the Taqman^TM^ Gene Expression Assay protocol. All samples were run in triplicate. The mRNA relative analysis expression was performed through the 2^−ΔΔCq^ method [31], and the glyceraldehyde 3-phosphate dehydrogenase (*GAPDH*) was used as a reference gene (Hs9999905_m1, Applied Biosystems).

### 2.8. Statistical Analysis

The statistical analyses were performed using STATA v 15 (College Station, TX, USA) and GraphPad Prism v 8.0 (San Diego, CA, USA). We evaluated the statistical power according to the calculation of sample size using the Fleiss formula for proportions in case-control studies [32]. According to the polymorphic frequencies of the *VDR* variants described in the González-Mercado study carried out in postmenopausal women from Western Mexico [33], we obtained a necessary sample size of at least 180 participants in each group to detect in the same number of alleles and risk genotypes described in the previous article in the Mexican population.

The Shapiro–Wilk test was used to determine the non-parametric and parametric distribution of the continuous variables. For the descriptive analysis, nominal variables were expressed as frequencies, continuous variables with parametric distribution were expressed as mean and standard deviation, and variables with non-parametric distribution were expressed as medians and percentiles from the 5^th^–95^th^. We determined the genotype and allele frequencies for the *VDR* gene variants by direct counting and performed the χ^2^ test to compare proportions between groups and to evaluate the Hardy–Weinberg equilibrium. To compare two groups, for parametric quantitative variables, we used the Student’s t-test, for non-parametric quantitative variables, we used the U Mann–Whitney test, and for more than two groups, we used the Kruskal–Wallis test.

We calculated the odds ratios (OR) with 95% confidence intervals (CI) to analyze the potential risk for SLE associated with the *VDR* gene variants. The linkage disequilibrium (LD) between *VDR* variants was expressed as Lewontin’s D’ corrected coefficient (D’) [34], and the haplotype frequencies were estimated using the expectation-maximization (EM) algorithm by Excoffier and Slatkin, both calculated with the SHEsis web tool [35,36].

We used logistic and linear regression models to evaluate the association between genotypes and haplogenotypes. For all analyses, the reference haplotype and haplogenotype were the most frequent. Differences were considered statistically significant at *p* < 0.05.

## 3. Results

### 3.1. Clinical Characteristics and Vitamin D Status in SLE and CS

In the present study, the SLE patients evaluated showed a median disease evolution time of 7 years, with 44% of clinical activity (Mex-SLEDAI ≥2), 34% of renal activity, 80% positivity to ANAs, and 43% to anti-dsDNA. The most administered drugs were prednisone (52%) and chloroquine (45%), while 20% reported the use of vitamin D supplements (8% cholecalciferol and 12% calcitriol) (Table 1).

Regarding the vitamin D status of the study groups, the median calcidiol was lower in SLE with 20.9 ng/mL (6.03 to 39.3) vs. CS with 23.1 ng/mL (10.9 to 39.4) (*p* = 0.03) with a frequency of calcidiol deficiency (<20 ng/mL) of 36% in SLE vs. 46% in CS without significant differences (*p* = 0.15). With respect to the calcitriol/calcidiol ratio, we observed a higher conversion of calcidiol to calcitriol in SLE with 2.16 pg/ng (0.73 to 10.5) vs. CS with 1.47 (0.33 to 4.31) (*p* < 0.001), reflected in higher calcitriol levels with 46.8 pg/mL (22.1 to 103) compared to CS with 36.8 pg/mL (9.64 to 82.6) (*p* < 0.001).

### 3.2. Genotypic and Allelic Frequencies of the VDR Variants in SLE vs. CS

The Hardy–Weinberg equilibrium of the *VDR* genetic variants was evaluated in the CS group and were observed in equilibrium in *FokI* (rs2228570) *p* = 0.77; *BsmI* (rs1544410) *p* = 0.68; *ApaI* (rs7975232) *p* = 0.85; *TaqI* (rs731236) *p* = 0.35.

We did not observe significant differences in the genotypic frequencies of the *VDR* variants between SLE patients vs. CS, and when determining different Ors, we also found no association with susceptibility to the disease. The most frequent genotypes for each *VDR* variants were the CT *FokI* genotype (SLE = 48% vs. CS = 51%; *p* = 0.32), GG *BsmI* genotype (SLE = 59% vs. CS = 59%; *p* = 0.99), AC *ApaI* genotype (SLE = 51% vs. CS = 48%; *p* = 0.77), and TT *TaqI* genotype (SLE = 56% vs. CS = 54%; *p* = 0.92) (Table 2).

Regarding allelic frequencies, we did not observe significant differences between the study groups. The most frequent alleles were the C *FokI* allele (SLE = 50% vs. CS = 55%; *p* = 0.20), G *BsmI* allele (SLE = 77% vs. CS = 77%; *p* = 0.99), C *ApaI* allele (SLE = 58% vs. CS = 57%; *p* = 0.98), and T *TaqI* allele (SLE = 75% vs. 75%; *p* = 0.77) (Table 2). 

### 3.3. Linkage Disequilibrium, Haplotypes, and Haplogenotypes of the VDR Variants in SLE and CS

**Figure 1:**
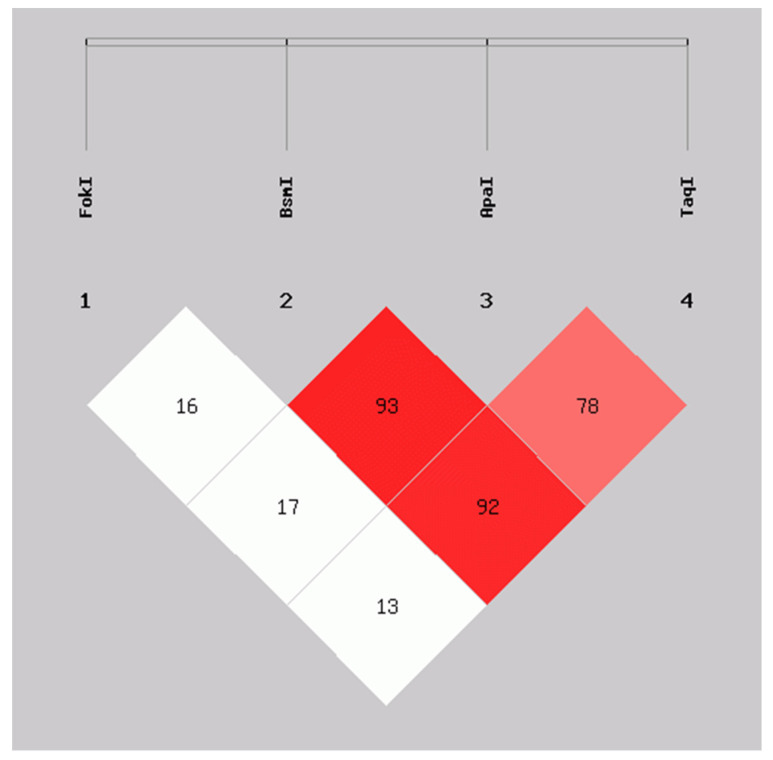
Linkage disequilibrium between FokI, BsmI, ApaI, and TaqI variants in VDR in SLE patients and CS. D’ values: FokI:BsmI = 0.16, FokI:ApaI = 0.17, FokI:TaqI = 0.13, BsmI:ApaI = 0.93, BsmI:TaqI = 0.92, ApaI:TaqI = 0.78. Calculated with SHEsis online software. SLE: systemic lupus erythematosus; CS: control subjects.

We evaluated the linkage disequilibrium (LD) expressed as Lewontin’s D’ coefficient (D’) between the *VDR* variants. *FokI* was not in LD with the *BsmI* (D’ = 0.16), *ApaI* (D’ = 0.17), and *TaqI* (D’ = 0.13) genetic variants. In contrast, high LD was observed between the *BsmI* and *ApaI* (D’ = 0.93), *BsmI* and *TaqI* (D’ = 0.92), as well as in *ApaI* and *TaqI* (D’ = 0.78) variants, which is indicative that *BsmI, ApaI*, and *TaqI* are segregating in blocks or haplotypes in this population studied (Figure 1).

Based on the previous findings, seven haplotypes of the *VDR* variants with high LD (*BsmI*, *ApaI*, and *TaqI*) present in the sample were inferred. From these, five haplogenotypes with a frequency greater than 5% were identified in the sample assessed, which were GCT/GCT, AAC/GAT, AAC/GCT, GAT/GCT, and GAT/GAT; as well as ten haplogenotypes with a frequency of less than 5%, which were grouped as “other haplogenotypes”, consisting of AAC/AAC, GAC/GCT, AAC/GCC, AAC/ACT, AAT/GCT, AAC/AAT, ACT/GCT, AAC/GAC, GCC/GCT, and GCC/GCC (Table 3).

We did not observe significant differences in the frequencies of the haplotypes (*p* = 0.94) and haplogenotypes (*p* = 0.71) between SLE and CS. The most frequent haplotypes were the GCT, with 53% in both groups, followed by the AAC (SLE = 22% vs. CS = 21%) and GAT (SLE = 21% vs. CS = 19%). The most frequent haplogenotypes were the GCT/GCT (SLE = 28% vs. CS = 30%) and the AAC/GCT (SLE = 24% vs. CS = 27%) (Table 3).

### 3.4. Vitamin D Status by FokI Genotypes and VDR Haplogenotypes in SLE Patients vs. CS

**Figure 2:**
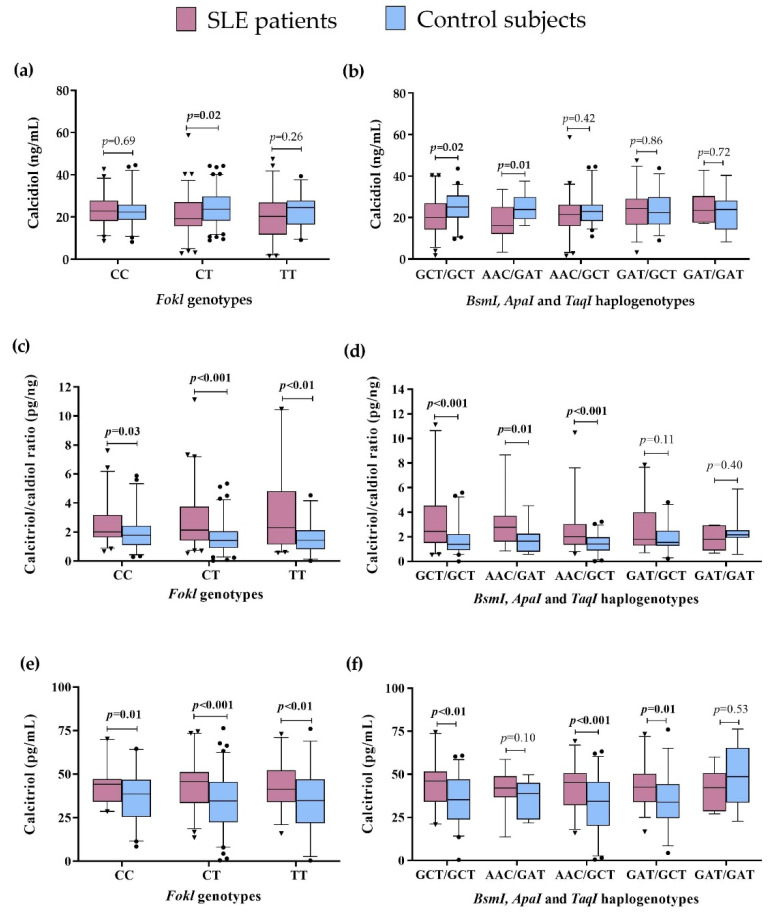
Vitamin D metabolism variables by VDR genotypes and haplogenotypes in SLE patients vs. CS. (**a**) Calcidiol serum levels by FokI genotypes in SLE vs. CS; (**b**) Calcidiol serum levels by BsmI, ApaI, and TaqI haplogenotypes in SLE vs. CS; (**c**) Calcitriol/calcidiol ratio by FokI genotypes in SLE vs. CS; (**d**) Calcitriol/calcidiol ratio by BsmI, ApaI, and TaqI haplogenotypes in SLE vs. CS, I Calcitriol serum levels by FokI genotypes in SLE vs. CS; (**f**) Calcitriol serum levels by BsmI, ApaI, and TaqI haplogenotypes in SLE vs. CS. Data provided in medians (p05th–p95th), Mann–Whitney test. Bold numbers mean significant differences (*p* < 0.05). SLE: systemic lupus erythematosus.

We compared the vitamin D serum levels to the *FokI* genotypes and *VDR* haplogenotypes in SLE patients vs. CS. Lower calcidiol levels were observed in SLE patients carrying the CT genotype (SLE = 19.2 ng/mL vs. CS = 23.6 ng/mL; *p* = 0.02) (Figure 2a), the GCT/GCT (SLE = 20.2 ng/mL vs. CS = 25.1 ng/mL, *p* = 0.02), and AAC/GAT haplogenotypes (SLE = 16.1 ng/mL vs. CS = 23.9 ng/mL, *p* = 0.01) (Figure 2b), while there was no difference in the presence of the TT, CC *FokI* genotypes, and other *VDR* haplogenotypes (Figure 2a,b, Table S1).

We observed a higher calcitriol/calcidiol ratio in SLE patients compared to CS, regardless of the *FokI* genotype they carried: CC (SLE = 2.01 pg/ng vs. CS = 1.78 pg/ng, *p* = 0.03), CT (SLE = 2.24 pg/ng vs. CS = 1.41 pg/ng, *p* < 0.001), and TT (SLE = 2.64 pg/ng vs. CS = 1.42 pg/ng, *p* < 0.01) (Figure 2c), and also independently of the *VDR* haplogenotypes: GCT/GCT (SLE = 2.43 pg/ng vs. CS = 1.39 pg/ng, *p*<0.001), AAC/GAT (SLE = 2.80 pg/ng vs. CS = 1.65 pg/ng, *p* = 0.01), and AAC/GCT haplogenotypes (SLE = 2.09 pg/ng vs. CS = 1.41 pg/ng, *p* < 0.001) (Figure 2d), with no significant differences in the other haplogenotypes evaluated (Figure 2d, Table S1).

Regarding the above, we also observed higher calcitriol serum levels in SLE compared to CS, independently of the *FokI* genotypes in the carriers of CC (SLE = 46.10 pg/mL vs. CS = 39.82 pg/mL; *p* = 0.001), CT (SLE = 47.9 pg/mL vs. CS = 35.2 pg/mL, *p* < 0.001), and TT (SLE = 50.8 pg/mL vs. CS = 34.92 pg/mL, *p* < 0.01) genotypes (Figure 2e). Moreover, carriers of the GCT/GCT (SLE = 48.6 pg/mL vs. CS = 38.1, *p* < 0.01), AAC/GCT (SLE = 46.1 pg/mL vs. 34.4 pg/mL, *p* < 0.001), and GAT/GCT (SLE = 47.1 pg/mL vs. CS = 36.5 pg/mL, *p* = 0.01) haplogenotypes showed higher calcitriol serum levels in SLE compared to CS (Figure 2f). No differences in the other haplogenotype carriers were observed (Figure 2f, Table S1).

These results highlight that the differences observed in the vitamin D serum status are not influenced by the *VDR* haplogenotype presence in SLE patients and CS. 

Additionally, we performed the analysis of these variables intra group. In SLE patients, we observed lower calcidiol serum levels in the presence of the AAC/GAT haplogenotype vs. GAT/GCT (*p* = 0.04). Concerning the CS, the AAC/GCT haplogenotype carriers vs. GAT/GAT showed a lower calcitriol/calcidiol ratio (*p* = 0.03) and lower calcitriol levels (*p* < 0.01) (Tables S1 and S2).

### 3.5. Association of the FokI Genotypes with the Vitamin D Status and Clinical Variables in SLE

In addition, we compared serum vitamin D status according to the *FokI* genotype within each study group. In the CS group, we did not observe significant findings, so in this section, the focus is on SLE. We observed lower calcidiol levels (19.1 ng/mL) and a higher frequency of calcidiol deficiency (53%) in the SLE patients carrying the CT *FokI* genotype, compared to other *FokI* genotypes, with non-significant differences. Concerning the calcitriol/calcidiol ratio, the SLE patients carrying the TT *FokI* genotype showed a significantly higher frequency in the 3rd tertile (53%; *p* < 0.01) compared to other genotypes (CT = 48% and CC = 38%; *p* < 0.01), while we did not observe a significant difference in the calcitriol tertiles (Table 4).

Regarding the clinical characteristics, we observed a lower Mex-SLEDAI score in the SLE patients carrying the CT *FokI* genotype compared to patients with the other *FokI* genotypes (Mex-SLEDAI: CC = 1.5 vs. CT = 0 vs. TT = 2; *p* = 0.03) (Table 4). Moreover, the group of the CT *FokI* genotype showed higher values of weight (CC = 62.8 kg vs. CT = 68.4 kg vs. TT = 67.5 kg; *p* = 0.04) and BMI (CC = 25.4 kg/m^2^ vs. CT = 28.4 kg/m^2^ vs. TT = 27.3 kg/m^2^, *p* = 0.02), compared to other genotypes (Table 4).

**Figure 3:**
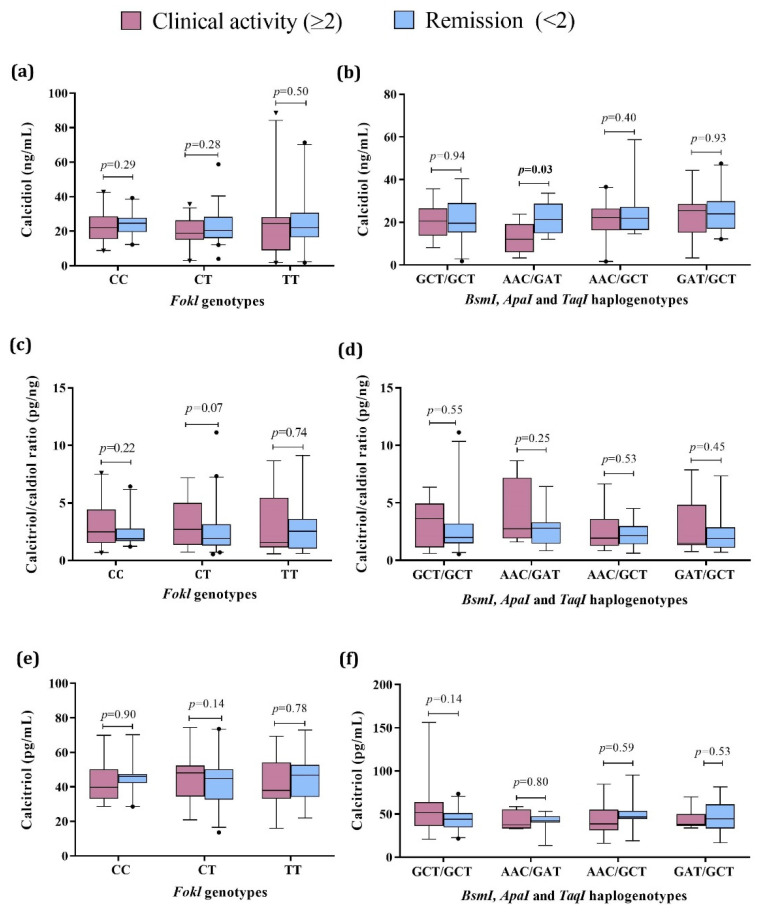
Vitamin D metabolism variables by VDR genotypes and haplogenotypes in active vs. remission SLE patients. (**a**) Calcidiol serum levels by FokI genotypes in SLE by clinical activity; (**b**) Calcidiol serum levels by BsmI, ApaI, and TaqI haplogenotypes in SLE by clinical activity; (**c**) Calcitriol/calcidiol ratio by FokI genotypes in SLE by clinical activity; (**d**) Calcitriol/calcidiol ratio by BsmI, ApaI, and TaqI haplogenotypes in SLE by clinical activity; (**e**) Calcitriol serum levels by FokI genotypes in SLE by clinical activity; (**f**) Calcitriol serum levels by BsmI, ApaI, and TaqI haplogenotypes in SLE by clinical activity. Data provided in medians (p05th–p95th), Mann–Whitney test. Bold numbers mean significant differences (*p* < 0.05). SLE: systemic lupus erythematosus Clinical activity (Mex-SLEDAI ≥ 2), Remission (Mex-SLEDAI < 2).

Further, we compared the vitamin D status in SLE patients considering both the presence of clinical activity by the Mex-SLEDAI index and the *VDR* genotypes and haplogenotypes. Concerning calcidiol, we did not observe differences by *FokI* genotypes (Figure 3a). At the same time, by the *VDR* haplogenotypes, the SLE group with the AAC/GAT haplogenotype and clinical activity showed lower serum calcidiol (12.3 ng/mL, *p* = 0.03) compared to the SLE in remission carriers of the same haplogenotype (21.4 ng/mL) (Figure 3b). No differences were observed in the calcitriol/calcidiol ratio or serum calcitriol levels for *VDR* genotypes and haplotypes (Figure 3c–f, Table S3).

In addition, we made the comparison according to renal activity. We did not observe significant differences in the calcidiol and calcitriol levels by the *FokI* genotypes and *VDR* haplogenotypes; regarding the calcitriol/calcidiol ratio, it was higher in patients with renal activity who were carriers of the CT *FokI* genotype, compared to those who did not present renal activity and were carriers of the same genotype (Table S4 and Figure S1).

### 3.6. VDR mRNA Expression in SLE Patients and CS by Vitamin D Status, Clinical Activity, and VDR Variants

**Figure 4:**
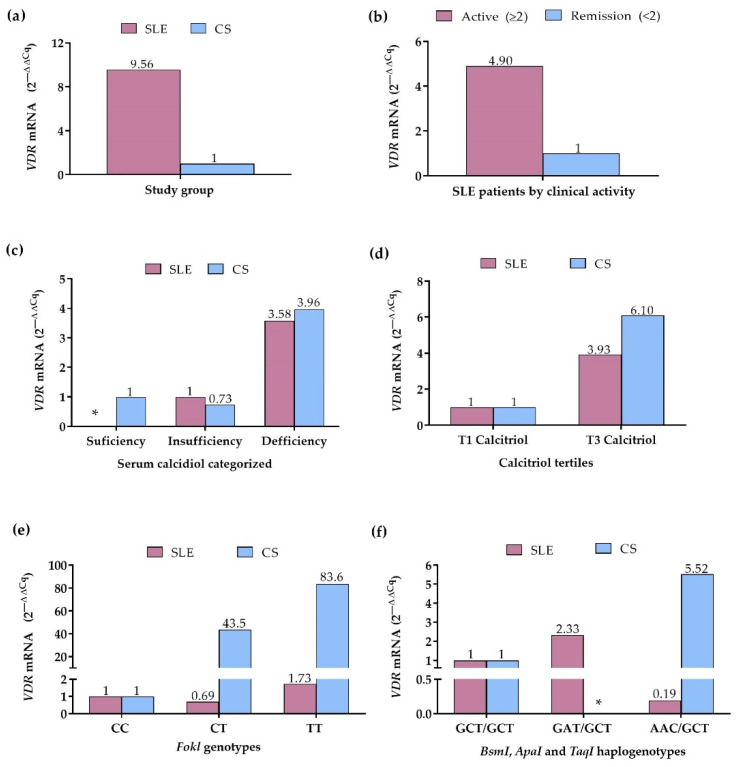
VDR mRNA expression in SLE patients and CS. (**a**) VDR expression in SLE patients vs. CS (CS: reference group); (**b**) VDR expression in SLE patients by clinical activity (Remission: reference group); (**c**) VDR expression in SLE patients and CS by calcidiol categorized (Sufficiency: reference group); (**d**) VDR expression in SLE patients and CS by calcitriol tertiles (T1: reference grI); (**e**) VDR expression in SLE patients and CS by FokI genotypes (CC genotype: reference group); (**f**) VDR expression in SLE patients and CS by BsmI, ApaI, and TaqI haplogenotypes (GCT/GCT haplogenotype: reference group. mRNA expression. Reference group = 1. 2 −∆∆Cq Method. SLE: systemic lupus erythematosus. CS: control subjects. *: without data.

Because the *VDR* expression can be influenced by genetic factors, such as the *VDR* variants, as well as by its ligand, we evaluated the mRNA *VDR* expression in peripheral blood leukocytes according to serum vitamin D status, *FokI* genotypes, *VDR* haplogenotypes, as well as according to clinical activity in the SLE group. In the comparison by study group, SLE patients showed a 9.56-fold higher *VDR* expression compared to CS (Figure 4a). In contrast, in SLE patients by clinical activity, the active patients showed 4.90-fold higher *VDR* expression compared to the remission group (Figure 4b). 

According to the categorized calcidiol levels, SLE patients with calcidiol deficiency showed 3.58-fold higher *VDR* expression compared to SLE patients with insufficiency; in the CS with calcidiol insufficiency, 1.36-fold lower *VDR* expression was observed (2^ΔΔCq^ = 0.73), whereas CS with deficiency showed 3.96-fold higher *VDR* expression, compared to CS with calcidiol sufficiency (Figure 4c). Complementary to the above, in the comparison by the calcitriol tertiles, 3.93-fold higher *VDR* expression was observed in SLE patients within the 3rd tertile, and in CS within the 3rd tertile, a 6.10-fold higher *VDR* expression was observed, compared to 1st tertile groups (Figure 4d).

In the analysis by the *FokI* variant, the CC genotype group of SLE and CS was considered the reference to assess the change in expression of the CT and TT genotypes. In SLE patients, *VDR* expression was 1.44-fold lower (2^ΔΔCq^ = 0.69) in CT carriers and 1.73-fold higher in TT carriers, while in CS, the expression was 43.5-fold higher in CT and 83.6-fold higher in TT genotype carriers, compared to the CC genotype group (Figure 4e). 

By the *VDR* haplogenotypes, the most frequent haplogenotype in SLE and CS, GCT/GCT, was taken as the reference and compared with the GAT/GCT and AAC/GCT haplogenotypes, which also presented high frequencies in both groups. In SLE, the GAT/GCT haplogenotype carriers showed a 2.33-fold higher *VDR* expression, while the AAC/GCT carriers exhibited a 5.26-fold lower *VDR* expression compared to the GCT/GCT group; in the CS, the AAC/GCT haplogenotype carriers presented 5.52-fold higher *VDR* expression than the CS with the reference haplogenotype (Figure 4f).

## 4. Discussion

In this case-control study, we explored the associations of *VDR* gene variants (*FokI, BsmI, ApaI,* and *TaqI*) with susceptibility to SLE, vitamin D serum status, and *VDR* mRNA expression in Mexican-Mestizo women. Before performing the association analysis, we verified that the Hardy–Weinberg equilibrium principle was fulfilled in the control group, as representatives of the general population [37]. In our study, the *VDR* variants evaluated were in Hardy–Weinberg equilibrium in both study groups, which is indicative that the alleles of all variants evaluated were randomly segregated from one generation to another and the sample was representative of the population assessed. [37].

We did not observe significant differences in the genotypic and allelic frequencies of the four *VDR* variants evaluated between SLE patients and CS, nor association with genetic risk for SLE. The frequencies observed were similar to those reported in postmenopausal women with and without osteoporosis from Western Mexico [33]. In contrast, previous SLE studies have reported various inconsistent association results with the *VDR* variants. In Egypt, a case-control study showed that the CC (FF) *FokI* genotype (OR = 4.9) and C (F) allele (OR = 1.9); AG (Bb) *BsmI* genotype (OR = 2.5), AA (BB) genotype (OR = 5.9), and A (B) allele (OR = 2.3); AA (AA) *ApaI* genotype (OR = 2.8) were associated with genetic risk to SLE [38]. In another study in Bulgaria, the risk genotypes and alleles for SLE were the CT (Ff) and TT (ff) *FokI* genotypes (OR = 2.6) and T (f) allele (OR = 2.14); AG (Bb) and GG (bb) *BsmI* genotypes (OR = 2.7) and G (b) allele (OR = 2.0) [39]. Similarly in India, the CT (Ff) (OR = 2.80) and TT (ff) *FokI* genotypes (OR = 2.57) and T (f) allele (OR = 1.96); CT (Tt) *TaqI* genotype (OR = 2.07) and T (t) *TaqI* allele (OR = 1.60) were associated with SLE [21]. 

These differences observed between populations of different populations highlight the role of racial influence and genetic factors in the susceptibility to autoimmunity diseases such as SLE [40]. In the Mexican-Mestizo population, there is a more significant Amerindian component on the coasts and south of Mexico, while a more important Caucasian component is in western and northern Mexico [41]. Our population assessed has a genetic background of Amerindian (21–25%), Caucasian (60–64%), and African (15%) ancestry, which gave origin to the Mexican-Mestizo population [41]. These results are evidence that the genetic structure of our Mexican-Mestizo population could mark differences in the frequency distribution of the *VDR* variants and its potential association with SLE or SLE clinical manifestations compared to other SLE populations.

Linkage disequilibrium is a valuable analysis that allows the location of disease-related genetic variants [37]. In the Mexican-Mestizo population evaluated in the present study, the *BsmI*, *ApaI*, and *TaqI* genetic variants in *VDR* presented a strong linkage disequilibrium (LD > 0.78), while the *FokI* variant was not in LD with these *VDR* variants. A strong LD indicates that their alleles are segregated in a block (called a haplotype) from one generation to another and may confer a similar risk [40]. Similar to our study, in various populations, such as Caucasian subjects with liver cirrhosis [42], children with lupus nephritis from Colombia [43], and postmenopausal women from western Mexico [33], the *Bsml*, *Apal*, and *Taql* have been described with strong linkage disequilibrium. In contrast, *Fokl* is on a low DL with the other *VDR* variants [33,42,43].

A haplotype is a group of genetic variants within an organism inherited from a single parent [44]. A diploid individual inherits a copy from each parent, so the combinations between genotypes would be called haplogenotype. Therefore, we decided to analyze haplotypic and haplogenotypic frequencies in the evaluated population. Regarding this, we did not observe significant differences between the SLE and CS groups, with a higher frequency of GCT/GCT, AAC/GCT, and GAT/GCT in both. Although many studies have addressed the relationship between *VDR* gene individual variants and the vitamin D status and SLE risk, few have analyzed this in terms of haplotypes or haplogenotypes. In previous studies in Egypt, the cAC (or FBa, in its reported form with the initial letter of the *FokI*, *BsmI,* and *ApaI* variants; OR = 2.5) and also CAA (or FBA; OR = 6.5) haplotypes were associated with SLE [38], while in another study the fb haplotype showed a higher frequency in SLE patients [45]. Furthermore, there is no consensus on the nomenclature to refer to the alleles of the *VDR* genetic variants. In some studies, authors used the first letter of the variant’s name in uppercase (ancestral allele) or lowercase polymorphic allele), while in others, such as in our study, the authors used the initial of the nucleotides involved in the change in the nucleotide sequence. 

Concerning other studies, in Egyptian [17,46] and Brazilian SLE patients [47] the FF *FokI* genotype compared to the ff genotype was associated with lower calcidiol levels. Our study observed an association of the CT *FokI* genotype with lower calcidiol levels in SLE patients compared to CS. In contrast, higher calcitriol/calcidiol ratio and higher calcitriol levels were observed in SLE compared to CS, independently of the *FokI* genotypes. This phenomenon indicates that the presence of genetic variants may influence the presence of low calcidiol levels in SLE, while the high conversion of calcidiol to calcitriol, as well as the higher calcitriol levels observed in SLE, could be due to other factors, such as the inflammatory process and exacerbation of the autoimmune response that patients experience. Various extrarenal cells, such as activated immune system cells, can produce calcitriol [48]. The activation of B cells induces the expression of the 1α-hydroxylases enzyme (*CYP27B1*), which leads to the synthesis and increase in calcitriol levels [12,48]. In other inflammatory diseases, such as asthma and chronic obstructive pulmonary disease (COPD), vitamin D metabolism may be deregulated by inflammatory processes. In these situations, there is an increase in cytokines, such as TNF-α, IL-1β, and TGF-β, which can induce the expression of the *CYP27B*; additionally, higher values of the calcitriol/calcidiol ratio and calcitriol levels have been observed in patients with these diseases compared to healthy subjects [49], similar to the pattern observed in our study where patients with SLE also have an inflammatory process.

We observed that SLE patients carrying the GCT/GCT haplogenotype presented lower calcidiol levels and a higher conversion of calcidiol to calcitriol, reflected in high calcitriol levels, compared to CS. In addition, we observed a lower serum calcidiol in the presence of the AAC/GAT haplogenotype, a higher calcitriol/calcidiol ratio in the presence of AAC/GAT and AAC/GCT, as well as higher calcitriol levels in the presence of AAC/GCT and GAT/GCT haplogenotypes in SLE patients vs. CS. No previous studies report similar results regarding analyzing serum vitamin D by the *VDR* haplogenotypes.

In the comparison within the SLE group, we did not observe significant differences between the calcidiol and calcitriol levels by the *FokI* genotypes. However, a higher frequency in the tertile 3 of the calcitriol/calcidiol ratio was observed in patients carrying the TT *FokI* genotype. In previous studies, in Egyptian SLE patients, higher SLEDAI scores were observed in carriers of the CC (FF) *FokI* and AA (BB) *BsmI* genotypes (*p* < 0.05) of the CAA (FBA) haplotype ( *p*< 0.001) [38], as well as in carriers of the CC (FF) *FokI* genotype in other Egyptian SLE populations [17,46]. Interestingly, the present study observed the CT *FokI* genotype associated with lower clinical activity in SLE patients. The CC and TT genotypes were associated with higher clinical activity assessed by the Mex-SLEDAI, while for *BsmI*, *ApaI*, and *TaqI* genetic variants we did not find significant differences. Subsequently, based on this finding, we analyzed serum vitamin D status according to clinical activity together with the *VDR* genotypes and haplogenotypes, and we did not observe an association.

The relationship observed in our study and others regarding the CC (FF) *FokI* genotype with the clinical disease activity in SLE, may be because of functionality. This genetic variant (the presence of the C or F allele) generates a new start codon (ATG) 9 bases pairs after the common starting site, which translates to a shorter VDRA protein of 424 amino acids instead of the wild-type full-length VDRA isoform of 427 amino acids [4,35]. The short VDRA isoform is somewhat more active than the long VDRA isoform in terms of its transactivation capacity as a transcription factor [4,36,37]. This greater transactivation capacity of the VDR in SLE patients could be causing this transcription factor to favor more polarization of the Th2 cell phenotype, which has been reported to be favored by calcitriol by inducing upregulation of GATA3 and STAT6 [12,50], and this cell profile plays a crucial role in the pathophysiology of SLE [51,52]. 

Additionally, we observed a higher BMI and weight in SLE patients carrying the CT *FokI* genotype. In this same group, we observed lower calcidiol levels than CS, and also a higher frequency of calcidiol deficiency, although not significant, whereby the excess weight present in those patients could influence this. Due to its fat-soluble characteristic, vitamin D can be stored in adipose tissue [53,54]. Subjects with normal weight and obesity can have similar vitamin D levels; however, in the presence of obesity, the compartments where calcidiol can be distributed, such as serum, muscle, fat, and liver, are increased [55].

When evaluating the *VDR* mRNA expression considering different categorical variables, we observed a higher *VDR* expression in SLE patients compared with CS. The *VDR* is expressed in different immune system cells, such as activated T and B lymphocytes, neutrophils, macrophages, and dendritic cells [12,56,57]; thus, its increased expression in SLE patients could be a reflection of hyperactivation of the immune system, as well as the presence of high levels of calcitriol observed in these patients since the VDR is a ligand-dependent receptor [12]. It has been described that the existence of multiple enhancers in the *VDR* gene locus may contribute to calcitriol-induced *VDR* expression [24]. 

To confirm those mentioned above, we made the comparison according to the calcidiol and calcitriol levels. In SLE and CS, we also observed a higher *VDR* expression in the presence of calcidiol deficiency and when we identified higher calcitriol levels represented by the tertile 3. The association between serum vitamin D status and *VDR* expression has not been described in the literature; however, other studies have evaluated VDR protein by immunoassays and flow cytometry. In a female BALB/c mouse model, others observed that the group with pristane-induced lupus that received 2 mg./kg/day of calcitriol showed a positive correlation between the VDR protein expression with the IgG infiltrates in the hippocampus detected by immunoassay. This increase in the infiltrate indicates an inflammatory process stimulated by the VDR protein expression [58]. In the presence of high calcitriol levels, a greater uptake and processing of autoantigens could favor the autoreactive immune response; hence, the beneficial effect of vitamin D can be obtained in moderate doses [59].

Another remarkable finding is the higher *VDR* mRNA expression in SLE patients with clinical activity compared with in remission, which could be related to an inflammatory process in the disease. In China, other authors who determined VDR expression by flow cytometry found a similar result to our finding in SLE patients and healthy subjects. They reported that VDR protein expression on CD4+ T helper (Th) cells and their subsets, especially Th1, T regulatory (Treg), and T follicular helper cells (Tfh), was increased in SLE; additionally, VDR expression was positively associated with clinical disease activity and cell apoptosis in SLE patients [60]. 

We also observed higher *VDR* mRNA expression in SLE patients and CS carriers of the TT *FokI* genotype compared to those carrying the CC genotype. We hypothesize that this could be due to a compensatory mechanism since the CC *FokI* genotype contains the C (or F) allele associated with generating the short isoform of 424 amino acids of VDR. This isoform has a greater capacity for transactivation since it interacts more efficiently with the transcription factor TFIIB [22] compared to the 427 amino acid-long VDR isoform associated with the T (or f) allele or TT *FokI* genotype, which would be expressing the *VDR* mRNA in greater quantity to compensate its low transactivation activity. However, no study previously supports this hypothesis regarding the *VDR* expression by the *FokI VDR* genotypes in autoimmune or healthy conditions; therefore, this hypothesis must be taken with caution or as a perspective for future studies.

According to our results, the strength of the present study was that the sample of SLE patients and CS evaluated was homogeneous in the following characteristics: all participants were female, from the same geographic area, classified as the Mexican-Mestizo population with three ancestors in the same geographic region, and of similar age, which reduces the bias regarding environmental and genetic factors of ancestry that could influence the results. 

With the present study, we do not suggest causality between the variables evaluated; due to its cross-sectional design, it only provides information on a specific time point. Another limitation is that we only provided information about the global clinical disease activity and renal activity in our population; however, we were unable to assess the clinical activity of other specific organs. We have the prospect of conducting future studies where we evaluate the serum levels of VDR by ELISA and the different VDR isoforms by Western Blot to determine their possible association with the *VDR* variants and clinical variables and, furthermore, to evaluate the expression of immune system cells that express VDR and that play a key role in the pathophysiology of SLE. Therefore, further studies in SLE focused on these points will be necessary. These will help to support the clinical and translational nutrigenetic interventions with vitamin D supplementation according to the *VDR* genetic variants in subsequent studies conducted on patients with autoimmune diseases.

## 5. Conclusions

The *FokI* variant was not in linkage disequilibrium with *BsmI, ApaI*, and *TaqI VDR* variants in the Mexican-Mestizo population. SLE patient carriers of the TT *FokI* genotype showed higher clinical disease activity scores. Notably, the *VDR* mRNA expression was higher in SLE patients vs. CS, in active vs. inactive SLE patients, and in participants of both study groups with vitamin D deficiency, higher calcitriol levels, and TT *FokI VDR* genotype carriers. 

## Figures and Tables

**Table 1:** Clinical characteristics in SLE patients.

Variable	Value (*n* = 194)
**Clinical variables**	
Age (years) ^a^	38 (21–60)
Disease evolution time (years) ^a^	7 (0.7–21)
SLICC ^a^	0 (0–4)
Mex-SLEDAI ^a^	0 (0–8)
Activity (≥2) ^b^	44 (76/171)
Renal activity ^b^	34 (34/101)
Renal insufficiency ^b^	14 (13/91)
Glomerular filtration rate (GFR) ^a^	108 (38.2–134)
G1: Normal or high (≥90 mL/min) ^b^	74 (113/154)
G2: Slight decrease (60-89 mL/min) ^b^	18 (28/154)
G3a a G5 (45 a <15 mL/min) ^b^	8 (13/154)
Weight (kg) ^a^	66.8 (49.6–102)
BMI (kg/m^2^) ^a^	26.9 (19.3–37.5)
**Serum variables**	
ANAs ^b^	80 (117/147)
Anti-dsDNA ^b^	43 (69/162)
Serum calcium (mg/dL) ^c^	9.20 ± 0.51
Serum albumin (mg/dL) ^a^	3.95 (2.93–4.66)
Serum urea (mg/dL) ^a^	29.1 (16–83)
Serum creatinine (mg/dL) ^a^	0.7 (0.48–1.4)
Uric acid (mg/dL) ^c^	5.31 ± 1.50
CRP (mg/L) ^a^	5.0 (0.2–24.6)
BUN (mg/dL) ^a^	13.7 (7.53–38.6)
C3 (mg/dL) ^a^	114 (55.3–150)
C4 (mg/dL) ^c^	17.6 ± 10.6
**Vitamin D status**	
Calcidiol (ng/mL) ^a^	20.9 (6.03–39.3)
Calcidiol categorized % (n)	
Deficiency (<20 ng/mL) ^b^	46 (78/170)
Insufficiency (20 a <30 ng/mL) ^b^	38 (64/170)
Sufficiency (≥30 ng/mL) ^b^	16 (28/170)
Calcitriol (pg/mL) ^a^	46.8 (22.1–103)
Calcitriol/calcidiol ratio (pg/ng) ^a^	2.16 (0.73–10.5)
**Treatment prevalence % (n)**	
Prednisone ^b^	52 (95/181)
Chloroquine ^b^	45 (81/181)
Hydroxychloroquine ^b^	32 (58/181)
Azathioprine ^b^	36 (65/179)
Vitamin D supplements ^b^	20 (19/97)
Cholecalciferol (400 UI/día) ^b^	8 (8/96)
Calcitriol (0.25 µg/día) ^b^	12 (11/95)

^a^ Data provided in medians (percentile: p05th–p95th). ^b^ Data provided in percentages (n/total patients). ^c^ Data provided in mean ± SD. Mex-SLEDAI: Mexican Systemic Lupus Erythematosus Disease Activity Index; SLICC/ACR: Systemic Lupus International Collaborating Clinics/American College of Rheumatology; BMI: body mass index; ANAs: antinuclear antibodies; Anti dsDNA: double-stranded deoxyribonucleic acid antibodies; CRP: C-reactive protein.

**Table 2:** Genotypic and allelic frequencies of *VDR* genetic variants in SLE patients and CS.

*VDR* Genetic Variant	SLE % (*n* = 194)	CS % (*n* = 196)	*p* Value *	OR (CI 95%)	*p* Value **
***FokI (*** **rs2228570)*VDR***					
**Genotype**					
CC	26 (51)	29 (57)	0.32	0.68 (0.37–1.25)	0.18
CT	48 (92)	51 (100)		0.70 (0.41–1.20)	0.17
TT ^§^	26 (51)	20 (39)		1.46 (0.80–2.66)	0.18
**Allele**					
C	50 (194)	55 (214)	0.20	0.83 (0.62–1.11)	0.19
T ^§^	50 (194)	45 (178)		1.20 (0.89–1.60)	-
***BsmI*** **(rs1544410) *VDR***					
**Genotype**					
AA	4 (9)	5 (9)	0.99	1.00 (0.34–2.98)	0.99
AG	37 (71)	38 (72)		0.99 (0.64–1.54)	0.98
GG ^§^	59 (114)	59 (115)		0.99 (0.33–2.93)	0.99
**Allele**					
A ^§^	23 (89)	23 (90)	0.99	0.90 (0.63–1.26)	-
G	77 (299)	77 (302)		0.99 (0.70–1.41)	0.99
***ApaI*** **(rs7975232) *VDR***					
**Genotype**					
AA	17 (34)	19 (38)		0.95 (0.51–1.77)	0.87
AC	51 (99)	48 (93)	0.77	1.13 (0.70–1.82)	0.58
CC ^§^	32 (61)	33 (65)		1.04 (0.56–1.95)	0.87
**Allele**					
A	42 (167)	43 (169)	0.98	0.99 (0.74–1.33)	0.98
C ^§^	58 (221)	57 (223)		1.00 (0.74–1.34)	-
***TaqI*** **(rs731236) *VDR***					
**Genotype**					
CC	5 (10)	5 (10)	0.92	0.98 (0.35–2.74)	0.96
CT	38 (75)	41 (80)		0.92 (0.59–1.42)	0.69
TT ^§^	56 (108)	54 (106)		1.01 (0.36–2.85)	0.96
**Allele**					
C	25 (95)	25 (100)	0.77	0.95 (0.67–1.33)	0.77
T ^§^	75 (291)	75 (292)		1.04 (0.74–1.47)	-

Data provided in percentages and n, *χ^2^ test, Fisher’s exact test. ** *p* value from Odds ratios (OR). Names of the genotypes and alleles represented by the corresponding nucleotides of the respective *VDR* variant. § = Reference category; *VDR*: vitamin D receptor gene; SLE: systemic lupus erythematosus; CS: control subjects.

**Table 3:** Haplotypes and haplogenotypes of the BsmI, ApaI, and TaqI variants in VDR in SLE patients and CS.

*BsmI* (rs1544410), *ApaI* (rs7975232), and *TaqI* (rs731236) *VDR* Variants					
Variable	Study Group				
**Haplotypes**	**SLE** **% (*n* = 386)**	**CS** **% (*n* = 392)**	***p*** Value *	**OR (CI 95%)**	***p*** Value **
GCT ^§^	53 (206)	53 (211)	0.94	1	-
AAC	22 (83)	21 (84)		1.01 (0.69–1.47)	0.95
GAT	21 (81)	19 (76)		1.09 (0.74–1.60)	0.64
GCC	3 (12)	3 (10)		1.22 (0.47–3.25)	0.63
ACT	0.5 (2)	1 (2)		1.02 (0.07–14.2)	0.98
AAT	0.5 (2)	1.5 (4)		0.51 (0.04–3.62)	0.43
GAC	0 (0)	1.5 (5)		0.20 (0.00–1.85)	0.11
**Haplogenotypes**	**SLE** **% (*n* = 193)**	**CS** **% (*n* = 196)**	***p*** value *	**OR (CI 95%)**	***p*** value *
GCT/GCT ^§^	28 (55)	30 (58)	0.71	1	-
AAC/GAT	10 (19)	8 (15)		1.33 (0.57–3.12)	0.46
AAC/GCT	24 (46)	27 (52)		0.93 (0.52–1.66)	0.80
GAT/GCT	23 (44)	18 (36)		1.28 (0.69–2.38)	0.38
GAT/GAT	4 (8)	6 (13)		0.70 (0.23–2.03)	0.47
Others	11 (21)	11 (22)		1.00 (0.46–2.15)	0.98

Data provided in percentages and *n* *χ^2^ test, Fisher’s exact test. ** *p* value from Odds ratios (OR). ^§^: Reference category. Other haplogenotypes: those found in the sample with a frequency of ≤5%: AAC/AAC, GAC/GCT, AAC/GCC, AAC/ACT, AAT/GCT, AAC/AAT, ACT/GCT, AAC/GAC, GCC/GCT, and GCC/GCC. Names of the haplogenotypes represented by the corresponding nucleotides of each variant in *VDR*. GCT/GCT: taken as reference for OR calculations; SLE: systemic lupus erythematosus; CS: control subjects.

**Table 4:** Vitamin D metabolism and clinical variables according to FokI genotypes in SLE patients.

Variable	*FokI* Genotypes in SLE Patients			*p* Value
	CC (*n* = 51)	CT (*n* = 92)	TT (*n* = 51)	
**Vitamin D metabolism variables**				
Categorized calcidiol % (n)				
Deficiency (<20 ng/mL) ^a^	32 (15/46)	53 (42/79)	47 (21/45)	0.22
Insufficiency (20 to <30 ng/mL) ^a^	48 (22/46)	34 (27/79)	33 (15/45)	
Sufficiency (≥30 ng/mL) ^a^	20 (9/46)	13 (10/79)	20 (9/45)	
Calcitriol tertiles % (n)				0.09
T3 (>48.10 to 157 pg/mL) ^a^	31 (14/45)	45 (29/65)	56 (24/43)	
T2 (>33.94 to ≤48.1 pg/mL) ^a^	49 (22/45)	29 (19/65)	25 (11/43)	
T1 (0.33 to ≤33.9 pg/mL) ^a^	20 (9/45)	26 (17/65)	19 (8/43)	
Calcitriol/calcidiol ratio tertiles % (n)				**<0.01**
T3 (>2.26 to 23.6 pg/ng) ^a^	38 (17/45)	48 (31/64)	53 (23/43)	
T2 (>1.39 to ≤2.26 pg/ng) ^a^	51 (23/45)	28 (18/64)	16 (7/43)	
T1 (0.01 to ≤1.39 pg/ng) ^a^	11 (5/45)	24 (15/64)	30 (13/43)	
**Clinical variables**				
Mex-SLEDAI ^b^	1.5 (0–9)	0 (0–8)	2 (0–8)	**0.03**
SLICC/ACR-DI ^b^	1 (0–4)	0 (0–4)	0 (0–4)	0.38
GFR (mL/min) ^b^	109 (78.6–137)	106 (12.5–133)	100 (58.7–133)	0.12
Weight (kg) ^b^	62.8 (44.3–84.1)	68.4 (52–95.9)	67.5 (49.6–102)	**0.04**
BMI (kg/m^2^) ^b^	25.4 (18.2–33.7)	28.4 (20.3–37.5)	27.3 (19.6–38.3)	**0.02**
Categorized BMI % (n)				
Without excess weight (<24.9 kg/m^2^) ^a^	48 (23/48)	29 (25/85)	31 (15/48)	0.08
With excess weight (≥24.9 kg/m^2^) ^a^	52 (25/48)	71 (60/85)	69 (33/48)	

^a^ Data provided in percentages and n, *χ*^2^ test. ^b^ Data provided in medians (percentile: p05th–p95th), Kruskal–Wallis test. Bold numbers mean significant differences (*p* < 0.05). SLE: systemic lupus erythematosus. GFR: glomerular filtration rate. BMI: body mass index. Mex-SLEDAI: Mexican Systemic Lupus Erythematosus Disease Activity Index. SLICC/ACR-DI: Systemic Lupus International Collaborating Clinics/American College of Rheumatology damage index.

## Data Availability

Data used to support the findings of this study are available from the corresponding author upon reasonable request.

## Supplementary Materials

The following supporting information can be downloaded at: https://www.mdpi.com/article/10.3390/genes13112016/s1, Figure S1: Calcitriol/calcidiol ratio by *VDR* genotypes and haplogenotypes in SLE patients by clinical activity; Table S1: Vitamin D serum status between SLE patients vs. CS according the *FokI* genotypes and *VDR* haplogenotypes; Table S2: Vitamin D serum status analysis intra each group (SLE patients and CS) by the *FokI* genotypes and *VDR* haplogenotypes; Table S3: Vitamin D serum status in SLE patients by clinical activity according the *FokI* genotypes and *VDR* haplogenotypes; Table S4: Vitamin D metabolism variables and *VDR* variants in SLE patients stratified by renal activity.
