# Supplementary material for: Vitamin D Receptor (VDR) Genetic Variants: Relationship of FokI Genotypes with VDR Expression and Clinical Disease Activity in Systemic Lupus Erythematosus Patients
Source: Genes (Basel). 2022 Nov 3;13(11):2016. doi: 10.3390/genes13112016 (PMC9689830; doi:10.3390/genes13112016)
Supplement: Supplementary file 1 [file genes-13-02016-s001.zip › genes-1964739-supplementary.pdf]

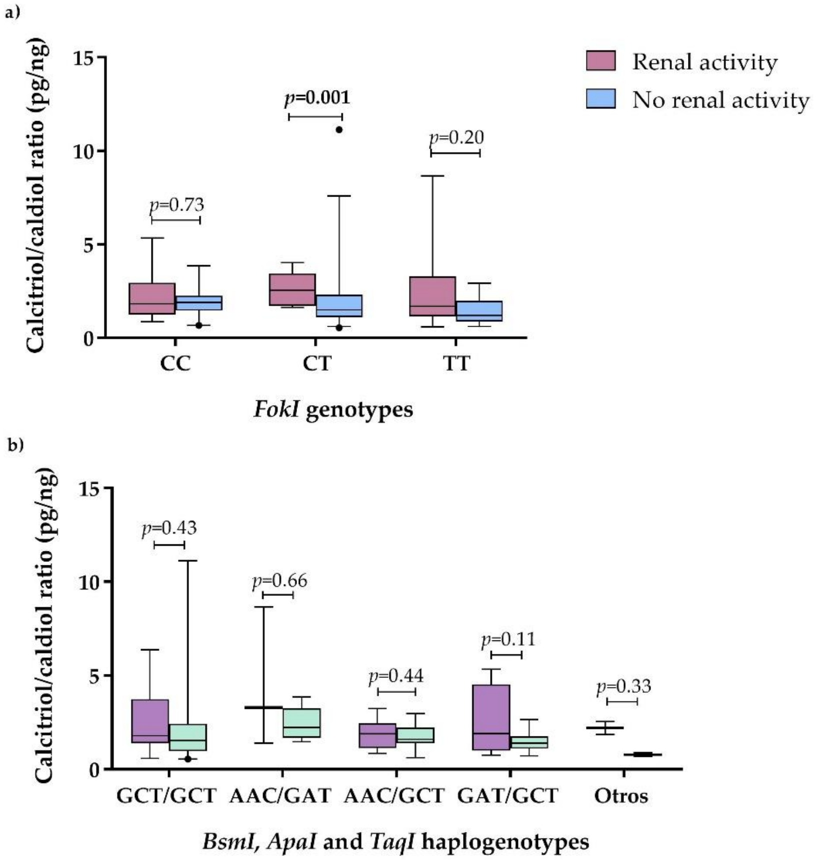


**Figure S1.** Calcitriol/calcidiol ratio by *VDR* genotypes and haplogenotypes in SLE patients by clinical activity. **a)** Calcitriol/calcidiol ratio by *FokI* genotypes in SLE, **b)** Calcitriol/calcidiol ratio by *BsmI*, *ApaI* and *TaqI* haplogenotypes in SLE. Data provided in medians ($p05^{th}$-$p95^{th}$), Mann-Whitney test. Highlighted values represent significant difference ($p<0.05$). SLE: systemic lupus erythematosus.

**Table S1.** Vitamin D serum status between SLE patients *vs.* CS according the *FokI* genotypes and *VDR* haplogenotypes

| Study group SLE *vs.* CS | Calcidiol (ng/mL) | | | Calcitriol/calcidiol ratio (pg/ng) | | | Calcitriol (pg/mL) | | |
|---|---|---|---|---|---|---|---|---|---|
| | Median | n | *p* value | Median | n | *p* value | Median | n | *p* value |
| *FokI* CC-SLE | 23.3 | 45 | 0.69 | 2.01 | 45 | **0.03** | 46.1 | 45 | **0.001** |
| *FokI* CC-CS | 22.3 | 57 | | 1.78 | 46 | | 39.8 | 46 | |
| *FokI* CT- SLE | 19.2 | 78 | **0.02** | 2.24 | 63 | **<0.001** | 47.9 | 64 | **<0.001** |
| *FokI* CT- CS | 23.6 | 100 | | 1.42 | 88 | | 35.2 | 88 | |
| *FokI* TT- SLE | 20.9 | 45 | 0.26 | 2.64 | 43 | **<0.01** | 50.8 | 43 | **<0.01** |
| *FokI* TT- CS | 24.4 | 38 | | 1.42 | 9 | | 34.9 | 29 | |
| GCT/GCT- SLE | 20.2 | 48 | **0.02** | 2.43 | 44 | **<0.001** | 48.6 | 44 | **<0.01** |
| GCT/GCT- CS | 25.1 | 56 | | 1.39 | 49 | | 38.1 | 49 | |
| AAC/GAT- SLE | 16.1 | 18 | **0.01** | 2.80 | 17 | **0.01** | 46.3 | 17 | 0.10 |
| AAC/GAT- CS | 23.9 | 15 | | 1.65 | 14 | | 39.3 | 14 | |
| AAC/GCT- SLE | 21.9 | 43 | 0.42 | 2.09 | 36 | **<0.001** | 46.1 | 37 | **<0.001** |
| AAC/GCT- CS | 23.0 | 52 | | 1.41 | 45 | | 34.4 | 45 | |
| GAT/GCT- SLE | 24.4 | 37 | 0.86 | 1.86 | 36 | 0.11 | 47.1 | 36 | **0.01** |
| GAT/GCT- CS | 22.3 | 35 | | 1.55 | 32 | | 36.4 | 32 | |
| GAT/GAT- SLE | 23.5 | 7 | 0.72 | 1.80 | 7 | 0.40 | 42.2 | 7 | 0.53 |
| GAT/GAT- CS | 23.9 | 11 | | 2.17 | 9 | | 48.7 | 9 | |

Data provided in medians (percentile: p05$^{th}$-p95$^{th}$), Mann-Whitney test. SLE: systemic lupus erythematosus. CS: control subject.

**Table S2.** Vitamin D serum status analysis intra each group (SLE patients and CS) by the FokI genotypes and VDR haplogenotypes

| Variable and study group | *FokI* genotypes *p* values | | | *VDR* haplogenotypes *p* values | | | | | | | | | |
|---|---|---|---|---|---|---|---|---|---|---|---|---|---|
| | CC *vs.* CT | CC *vs.* TT | CT *vs.* TT | GCT/GCT *vs.* AAC/GAT | GCT/GCT *vs.* AAC/GCT | GCT/GCT *vs.* GAT/GCT | GCT/GCT *vs.* GAT/GAT | AAC/GAT *vs.* AAC/GCT | AAC/GAT *vs.* GAT/GCT | AAC/GAT *vs.* GAT/GAT | AAC/GCT *vs.* GAT/GCT | AAC/GCT *vs.* GAT/GAT | GAT/GCT *vs.* GAT/GAT |
| **SLE patients (intra group)** | | | | | | | | | | | | | |
| Calcidiol (ng/mL) | 0.11 | 0.22 | 0.88 | 0.21 | 0.61 | 0.38 | 0.30 | 0.17 | **0.04** | 0.08 | 0.50 | 0.41 | 0.59 |
| Calcitriol/calcidiol ratio (pg/ng) | 0.95 | 0.65 | 0.43 | 0.84 | 0.44 | 0.87 | 0.22 | 0.71 | 0.90 | 0.43 | 0.46 | 0.45 | 0.27 |
| Calcitriol (pg/mL) | 0.86 | 0.50 | 0.58 | 0.61 | 0.43 | 0.53 | 0.20 | 0.22 | 0.25 | 0.13 | 0.97 | 0.39 | 0.46 |
| **Control subjects (intra group)** | | | | | | | | | | | | | |
| Calcidiol (ng/mL) | 0.48 | 0.49 | 0.78 | 0.90 | 0.15 | 0.13 | 0.49 | 0.42 | 0.27 | 0.61 | 0.52 | 0.89 | 0.96 |
| Calcitriol/calcidiol ratio (pg/ng) | 0.26 | 0.59 | 0.75 | 0.94 | 0.17 | 0.74 | 0.21 | 0.34 | 0.86 | 0.22 | 0.32 | **0.03** | 0.20 |
| Calcitriol (pg/mL) | 0.09 | 0.95 | 0.17 | 0.82 | 0.33 | 0.55 | 0.05 | 0.34 | 0.90 | 0.15 | 0.09 | **<0.01** | 0.09 |

The *p* values indicate the differences found within each group. Mann-Whitney test. The data of the medians of each group is found in table S1

**Table S3.** Vitamin D serum status in SLE patients by clinical activity according the *FokI* genotypes and *VDR* haplogenotypes

| Study group by clinical activity | Calcidiol (ng/mL) | | | Calcitriol/calcidiol ratio (pg/ng) | | | Calcitriol (pg/mL) | | |
|---|---|---|---|---|---|---|---|---|---|
| | **Median** | **n** | ***p* value** | **Median** | **n** | ***p* value** | **Median** | **n** | ***p* value** |
| FokI CC-Active SLE | 21.9 | 20 | 0.29 | 2.48 | 20 | 0.22 | 46.6 | 20 | 0.90 |
| FokI CC- Remission SLE | 24.5 | 22 | | 1.90 | 21 | | 46.2 | 21 | |
| FokI CT- Active SLE | 18.9 | 26 | 0.28 | 3.40 | 20 | 0.07 | 49.2 | 20 | 0.14 |
| FokI CT- Remission SLE | 20.3 | 45 | | 1.91 | 40 | | 45.4 | 41 | |
| FokI TT- Active SLE | 24.5 | 21 | 0.50 | 1.90 | 20 | 0.74 | 44.8 | 20 | 0.78 |
| FokI TT- Remission SLE | 21.9 | 20 | | 2.64 | 19 | | 50.8 | 19 | |
| GCT/GCT- Active SLE | 20.6 | 17 | 0.94 | 3.63 | 14 | 0.55 | 52.5 | 14 | 0.14 |
| GCT/GCT- Remission SLE | 19.6 | 29 | | 2.11 | 28 | | 45.7 | 28 | |
| AAC/GAT- Active SLE | 12.1 | 7 | **0.03** | 4.21 | 6 | 0.25 | 44.9 | 6 | 0.80 |
| AAC/GAT- Remission SLE | 21.4 | 11 | | 2.80 | 11 | | 46.3 | 11 | |
| AAC/GCT- Active SLE | 22.2 | 20 | 0.40 | 2.10 | 18 | 0.53 | 42.1 | 18 | 0.59 |
| AAC/GCT- Remission SLE | 22.9 | 17 | | 2.15 | 15 | | 46.7 | 16 | |
| GAT/GCT- Active SLE | 25.5 | 14 | 0.93 | 1.60 | 14 | 0.45 | 47.1 | 14 | 0.53 |
| GAT/GCT- Remission SLE | 23.9 | 20 | | 1.92 | 19 | | 44.7 | 19 | |
| GAT/GAT- Active SLE | 36.5 | 2 | 0.20 | -- | -- | -- | 48.0 | 7 | -- |
| GAT/GAT- Remission SLE | 20.6 | 3 | | -- | -- | | 46.7 | 7 | |

**Table S4.** Vitamin D metabolism variables and *VDR* variants in SLE patients stratified by renal activity.

| Variable | Renal activity (n=34) | No renal activity (n=69) | *p* value |
|---|---|---|---|
| **Vitamin D metabolism variables** | | | |
| Calcidiol (ng/mL) [a] | 20.6 (3.26-44.3) | 25.1 (12.2-40.4) | 0.13 |
| Calcitriol (pg/mL) [a] | 47 (19.0-53.2) | 41.5 (21.8-51.2) | **0.02** |
| Calcitriol/calcidiol ratio (pg/ng)[a] | 2.13 (0.76-15.3) | 1.54 (0.71-3.21) | **0.02** |
| ***VDR* variants** | | | |
| ***FokI* genotypes % (n)**[b] | | | 0.40 |
| CC | 21 (7/34) | 30 (21/69) | |
| CT | 47 (16/34) | 48 (33/69) | |
| TT | 32 (11/34) | 22 (15/69) | |
| ***BsmI* genotypes % (n)**[b] | | | 0.44 |
| AA | 6 (2/34) | 2 (1/69) | |
| AG | 35 (12/34) | 35 (24/69) | |
| GG | 59 (20/34) | 64 (44/69) | |
| ***ApaI* genotypes % (n)**[b] | | | 0.93 |
| AA | 15 (5/34) | 14 (10/69) | |
| AC | 47 (16/34) | 51 (35/69) | |
| CC | 38 (13/34) | 35 (24/69) | |
| ***TaqI* genotypes % (n)**[b] | | | 0.69 |
| CC | 3 (1/34) | 1 (1/69) | |
| CT | 44 (15/34) | 38 (26/69) | |
| TT | 53 (18/34) | 61 (42/69) | |

[a]Data provided in medians (percentile: p05th-p95th), Mann-Whitney test. [b]Data provided in percentages and n, $\chi 2$ test. Highlighted data indicate significant differences.
